# Doing Philosophy Effectively: Student Learning in Classroom Teaching

**DOI:** 10.1371/journal.pone.0137590

**Published:** 2015-09-17

**Authors:** Natascha Kienstra, Jeroen Imants, Machiel Karskens, Peter G. M. van der Heijden

**Affiliations:** 1 Graduate School of Education, Radboud University, Nijmegen, the Netherlands; 2 Department of Philosophy, Radboud University, Nijmegen, the Netherlands; 3 Department of Methodology and Statistics, Utrecht University, Utrecht, the Netherlands; 4 S3RI, University of Southampton, Southampton, United Kingdom; University of Westminster, UNITED KINGDOM

## Abstract

An important aim of teaching philosophy in Dutch secondary schools is to *learn* about philosophy (i.e., the great philosophers) by *doing* philosophy. We examined doing philosophy and focused specifically on the relationship between student learning activities and teacher behavior; in doing so, a qualitative cross-case analysis of eight philosophy lessons was performed. The effectiveness of doing philosophy was operationalized into five learning activities comprising rationalizing, analyzing, testing, producing criticism, and reflecting, and scored by means of qualitative graphical time registration. Using CA we find a quantitative one-dimensional scale for the lessons that contrasts lessons that are more and less effective in terms of learning and teaching. A relationship was found between teaching by teachers and doing philosophy by students. In particular we found students to produce a higher level of doing philosophy with teachers who chose to organize a philosophical discussion with shared guidance by the teacher together with the students.

## Introduction

Unlike in other countries, in the Netherlands secondary schools can choose to include philosophy as a distinct, optional secondary school subject from the tenth grade onward for pre-university and senior general higher education students [1]. An important aim in Dutch classroom teaching is to *learn* about philosophy by *doing* philosophy, an approach advocated by great philosophers such as Plato and Kant, which is the focus of this paper. The most important learning theory where the student ‘learns by doing’ is constructivism [2]. Learning is seen as a largely interactive process of constructing new knowledge and skills based on the information that is already available within a person [3]. Tradition sees philosophy as the subject that concerns itself with truth. For philosophers, “What is truth?” is among the most important philosophical questions [4]. However, in addition to contemplating this question, a method of truth finding must also be adopted.

Teachers can use various philosophical exercises for doing philosophy and truth finding. Kienstra, Karskens, and Imants [5] defined philosophical exercises as “…a complex standardized manner of doing philosophy, in which philosophical knowledge and skills are combined to exchange thoughts [while] paying explicit attention to philosophical (meta)concepts in a lifelike context.” In this respect, one person or several individuals come to realize that “…they are actually ignorant and subsequently continue to inquire on a metalevel with the aim of constructing a true belief.” ([5], p. 291). A survey of the literature identified 30 distinct exercises to support students to do philosophy [5]. Examples of such exercises include essay writing, a debate or Socratic dialogue, delivering a speech, and discussing dilemmas [6].

Philosophy teachers in the Netherlands seem to only use a limited number of philosophical exercises, with classroom talk as a commonly used one and preferred by 75% of Dutch philosophy teachers [1]. However, classroom talk is not necessarily the most adequate exercise given a teacher’s goals for a specific lesson [6], and superficial implementations of classroom exercises can be a potential concern [7]. In a review of secondary school teaching materials, such occurrences were encountered, wherein a limited number of distinct exercises were utilized that were often not of a philosophical nature, or lacked depth from a theoretical point of view [5].

It is unknown which philosophical exercises are effective at encouraging students to do philosophy at a high level. Therefore, we should determine to what extent students are engaged in doing philosophy in interaction with their teacher. We also consider philosophical and classroom context factors as conditions for the effectiveness of exercises.

**Fig 1:**
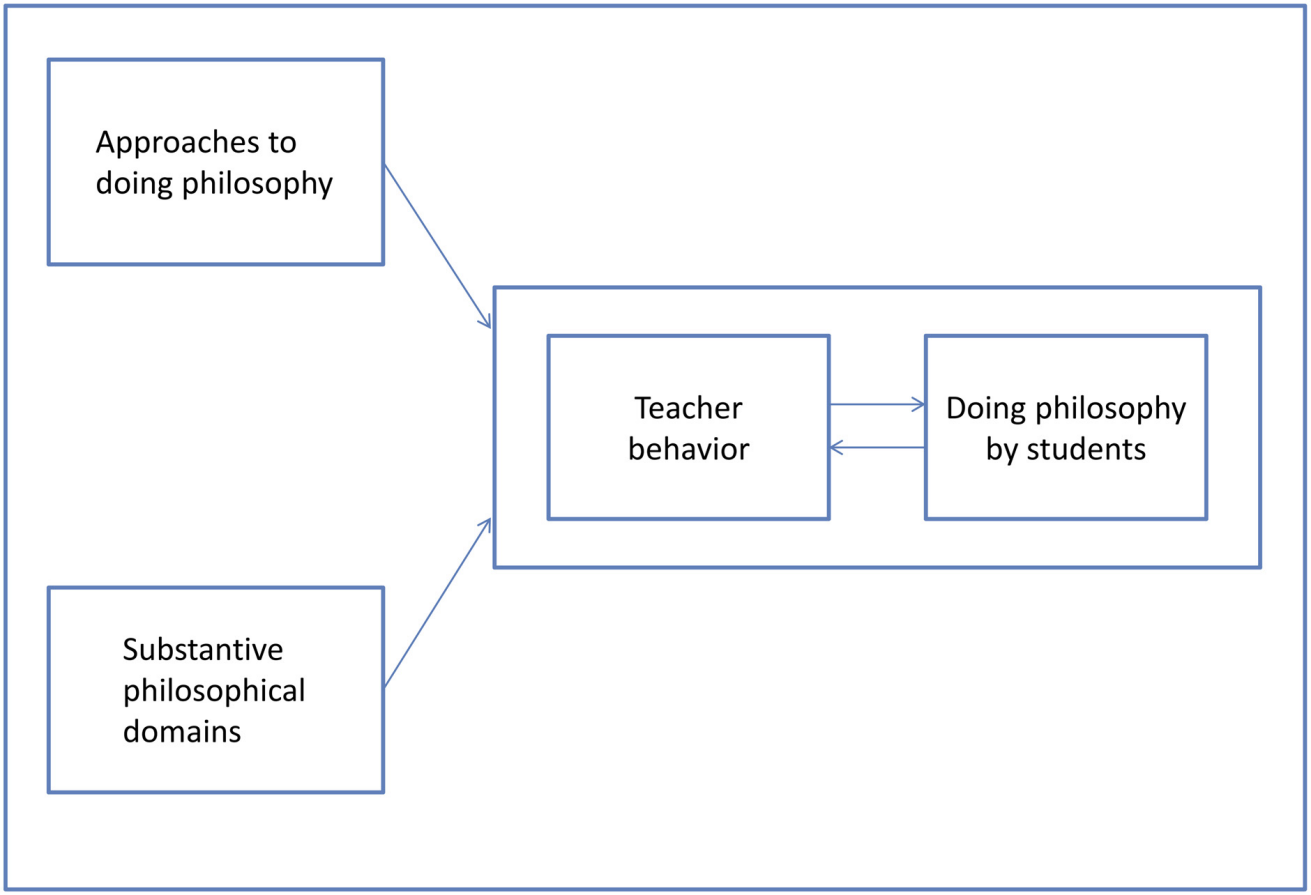
Conceptual framework. Students doing philosophy and teacher behavior in a philosophy lesson.

Fig 1 shows a conceptual framework of a philosophy lesson wherein the relation of teacher behavior with doing philosophy by students has a central role. The relationship between teacher behavior and doing philosophy by the students is influenced by the teacher’s design of the lesson.

The concepts in Fig 1 are related to the intended, implemented, and attained curriculum models [8], [9], in addition to the design, execution, learning activities, and results of learning [10]. A clustering of philosophical exercises that we refer to as approaches to doing philosophy and substantive philosophical domains such as ethics and philosophy of science are related to the intended curriculum and its design. Teacher behavior is related to the implemented curriculum and its execution. Furthermore, doing philosophy by students is related to the attained curriculum, learning activities, and learning results. The reciprocal relationship between teacher behavior and doing philosophy by students in Fig 1 is akin to the distinction between an instructor’s teaching and student learning found in output driven activities and the learning process. Here the activities of doing philosophy by students develop by means of interaction with the teacher. The four concepts presented in Fig 1 are elaborated upon below.

### Doing philosophy: A Pearl Model

What behaviors do students actually show when they do philosophy? A review of the literature pointed to five distinct and hierarchically ordered activities: (1) rationalizing, (2) analyzing, (3) testing, (4) producing criticism, and (5) reflecting ([5], compare [11], [12]). *Rationalizing* involves the verbalization of initial thoughts in a logical structure, while *analyzing* entails continuous questioning, wondering, interrogation, problematization, and consideration. *Testing* encompasses evaluation, establishing definitions and distinctions, and making judgments. The *production of criticism* involves reasoning based on explanations, causes, and connections, in addition to pro/con arguments, the construction and maintenance of logical arguments, and debate. Finally, *reflection* entails making metaremarks, mirroring, creative leaps, and thinking about the thought process itself, as well as reflecting on pro/con arguments, the assessment framework, and its application [13], [14].

**Fig 2:**
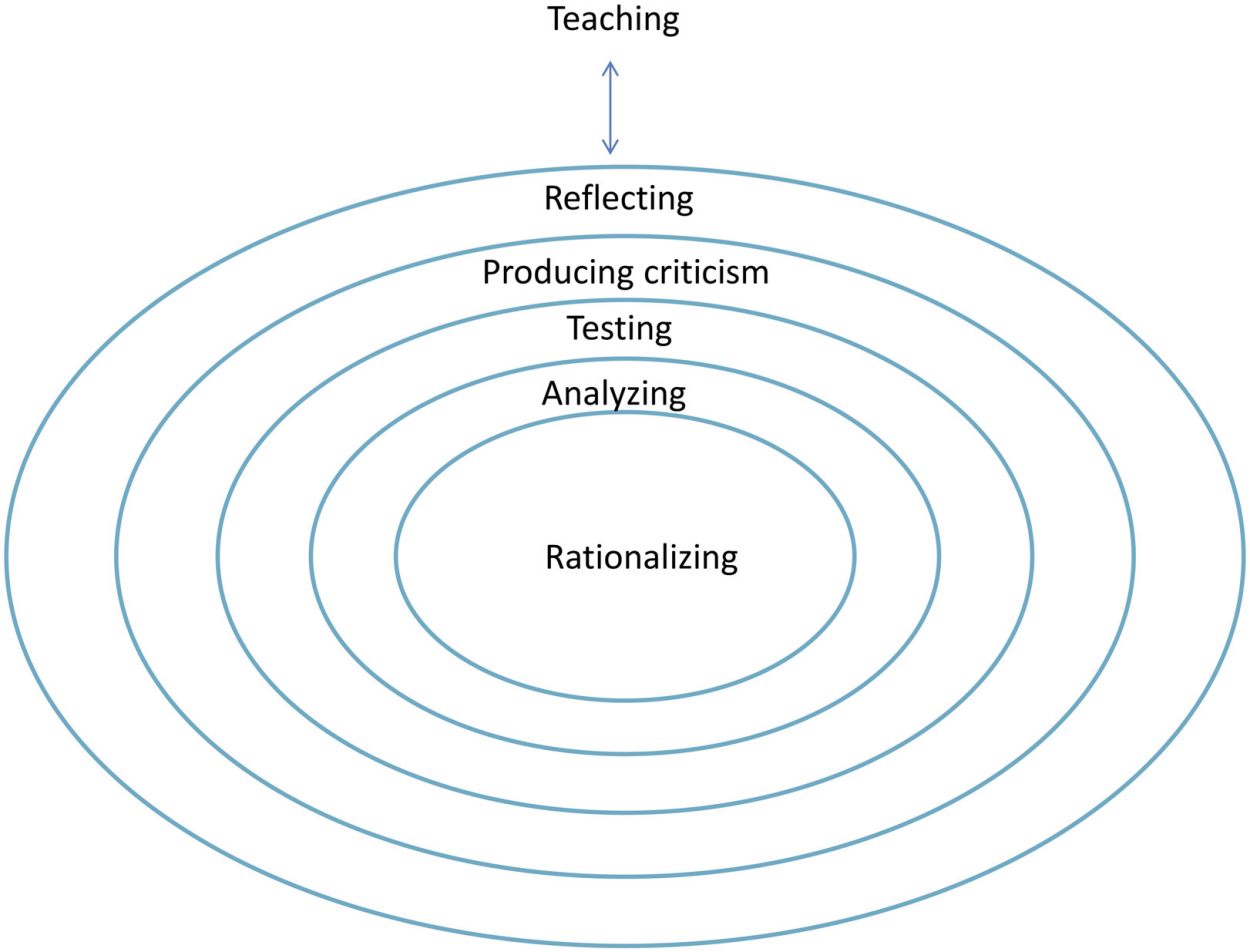
Pearl Model.

Doing philosophy occurs in phases during a lesson. In the Netherlands, a typical subject-specific lesson takes 50 minutes. Students cannot learn at their highest level for a whole lesson [15], [16]. Indeed, there will be moments wherein the levels of doing philosophy will be high and these levels will fluctuate due to social talk, organizational and classroom management conversations, and sidesteps. Classroom observation studies should integrate this understanding of the dynamic nature of lessons when analyzing the quality of education (teaching and learning). We describe a model of doing philosophy that we have dubbed the so-called Pearl Model (see Fig 2). In this model, pearls are composed of concentric layers that represent each of the five aforementioned activities. These activities are ordered hierarchically and conditionally. This indicates, for example, that while rationalizing exists at a lower level than reflecting, reaching the level of reflection assumes that rationalizing has also has taken place. Therefore, the higher the level that a pearl reached and the more layers have been reached, the more thorough the philosophical understanding, and the more effectiveness of doing philosophy are. Metaphorically, a pearl “shines” if the level of *reflection* has been reached while doing philosophy. It should be noted, however, that during interactions between a teacher and his or her students that both parties need not necessarily be at identical levels; it is possible, therefore, that a teacher might ask a question or present an idea from a layer that his or her students have not reached (see Fig 2).

Doing philosophy is perceived as an interactive group activity, and therefore we speak of doing philosophy in the classroom. Doing philosophy as *vita contemplativa* is outside our scope. Thus it is outside our scope what students think without saying it, what students learn *after* the lesson and what they understood. Also, to learn doing philosophy is understood as a process that ‘happens’ in a conversation. Thus it also remains outside our scope that learning is not necessarily (only) dependent on the dialogue in the lesson, but also from all sorts of other factors, inside, outside and between the lessons. Doing philosophy is thinking about how we use concepts and how concepts operate.

In the process of concept formation also the beliefs of others are represented in a(n inner) dialogue because incorporated adjustments of concepts are picked up, criticized and accepted (compare [17], p.151). Concept formation is dialogic of intent and as such is close to doing philosophy in classroom teaching.

In a philosophy lesson, students construct a common concept (i.e., answer) to a philosophical question [18]. In the present study, we operationalize common concept formation (CCF) by using Brüning’s [19] taxonomy of conceptual analysis, which includes (i) a deductive ladder leading from the abstract to concrete; (ii) the building of sentences by thinking aloud about how a concept can be used; (iii) defining; and (iv) searching for counterexamples/exploring boundaries. Therefore, at higher layers of doing philosophy, a more reflexive form of CCF is expected.

### Teacher behavior

In terms of a philosophy teacher’s practice, the form of working and way of guidance are particularly important. Teachers can vary in their teaching practice from open to more closed practices [20]. A philosophical discussion, for example, has a more open form than a classroom talk. We expect that higher levels of doing philosophy are encountered in more open educational contexts (e.g. in philosophical discussions more than during classroom talks, since students do philosophy while interacting with each other in a discussion by using different words, forming concepts, and making distinctions).

Guidance by a teacher when students are engaged in philosophical exercises can be provided in a strong, shared, or loose manner [14], [21]. When a teacher constantly provides input (i.e., he alone asks questions and provides answers), guidance can be regarded as strong; in contrast, if a teacher uses a learner centered teaching approach and leaves students much room to choose for example the topic of a lesson or the way in which that topic is addressed, guidance is considered loose. Besides, guidance can be considered shared when a teacher and the students engage in a common dialogue, and jointly contribute to the topics and questions. Such dialogues possess the characteristics of a so-called ideal pedagogical speech situation [22], wherein educational rather than indoctrinatory, non-reflexive teaching situations are encountered. Moreover, when guidance is shared, higher levels of doing philosophy are expected than when guidance is loose or strong.

Three philosophical teaching styles can be distinguish: problem oriented, historically oriented and person oriented [23]. According to the problem oriented teaching style doing philosophy means solving philosophical problems or finding answers to philosophical questions. According to the historical teaching style the most important task of the philosopher is interpreting and reinterpreting the philosophical past using existing philosophical texts. According to the person oriented teaching style, doing philosophy is an attempt to create an individual, reasonably justified worldview. Another conception of philosophy has greater consequences for a teacher’s practice than telling another story or reading other texts in the classroom, because “it implies a completely different atmosphere in the classroom, with another distribution of roles for the teacher and the students. In one classroom students work quietly on a problem while the teacher assigns them tasks, in another class the teacher provides a lively performance while fascinated students are listening, and in the third there may be a lively exchange of ideas” ([23], p.24). However, the authors do propose a combination of teaching styles given the demands of the curriculum, the students and philosophy itself. We expect that doing philosophy effectively will occur more easily when a teacher is able to combine more than one philosophical teaching styles in a lesson.

### Design of a lesson

Here we discuss approaches to doing philosophy and substantive philosophical domains (see Fig 1).

#### Approaches to doing philosophy

Kienstra et al. [5] found 30 philosophical exercises in the literature and a content analysis of these 30 exercises resulted in three distinct and typical approaches to doing philosophy:

1. Doing philosophy as a method of connective truth finding or communicative action.
2. Doing philosophy as a type of test-based truth finding.
3. Doing philosophy in the form of a juridical debate, which entails judging truth-value and making judgments (i.e., truth-value analysis).

The first approach involves doing philosophy as a form of connective truth finding, wherein students search for the truth collectively through narratives and conversations. The second approach entails doing philosophy as a kind of test-based truth finding, in which students search for scientific truth as practiced by scientists. Finally, the third approach involves doing philosophy as a juridical way of finding the truth and truth-value, by debating competing or opposite claims, after which a competent “judge” reaches a verdict [24]. In this study, we present these three approaches to philosophy as a relevant educational context in which teacher and student activities can be understood.

Classroom talk, the exercise preferred by most Dutch philosophy teachers [1], fits the criteria mentioned in the first approach. Given our expectation that lower levels of doing philosophy will manifest during classroom talk, we accordingly predict that higher levels of doing philosophy will occur less frequently in the first approach.

#### Substantive philosophical domains

A number of substantive philosophical domains are used to teach philosophy at secondary schools in the Netherlands [25], [26]. In this paper, a distinction is made between domains closer to students’ real life experiences (e.g., philosophical anthropology, ethics, and social philosophy) and more abstract domains (e.g., theory of knowledge and the philosophy of science). We assume that it is possible to do philosophy on a high level regardless of domain, although a specific substantive philosophical domain may require specific exercises [1]. Thus, a relationship between substantive domains and higher levels of doing philosophy could be affected by the exercise selected, assuming that it can be categorized into one of the three approaches to doing philosophy.

## Method

To study the relations between the teaching context and students’ activities a mixed-methods comparative case study methodology was adopted [27] in which complete lessons were compared. The number of lessons that we study is eight, and each of these eight cases is studied thoroughly, yielding a situation that the number of variables is larger than the number of cases. A comparative case study methodology implies a two-phase approach in which first each lesson is analyzed separately and second a comparison between the cases is made. For the comparison of cases Miles and Huberman [28] proposed a so-called “meta-matrix” as a tool in which the main findings for each of the cases are summarized. In this study, we combined a qualitative multiple case study with a quantitative analyses of correspondence across lessons. Therefore the descriptors of the lessons were translated in category quantifications. Correspondence analysis (CA) (see [29], for a recent overview) is a popular tool for the graphical representation of data, in particular categorical data, in a multidimensional space.

### Participants

The participants in these lessons included seven teachers (one female and six males) and their students. The number of students in class ranged from 10 to 25 (average 16.5). Eight philosophy lessons were examined in their entirety (one of the teachers taught two lessons). During the 2010–11 school year, the aforementioned teachers were enrolled in a continuing education course at the first authors’ university, which was intended to familiarize secondary school teachers with a newly introduced final exam topic. These eight lessons were not a part of the continuing education course, however.

Teachers were asked to utilize one philosophical exercise from among a list of 30 [5], and also told that the selected exercise should result in doing philosophy in the classroom. Three participants selected exercises from the Juridical debate approach (namely Sic et Non, thought experiment, and speech), while the teacher who taught two lessons adopted the Socratic method, which is classified as both a Connective truth finding and Test-based truth finding approach. The two remaining instructors selected brainstorming and classroom talk/presentation, which are considered Test-based truth finding and Connective truth finding approaches respectively.

We also collected data about teacher characteristics because it is likely that these also play a role in the interaction between teachers and students. Teachers were questioned prior to beginning their lessons: (i) if the goals of the teaching philosophy (i.e., to learn philosophy by doing philosophy) are usually achieved [30], [31] (‘yes’ or ‘no’); (ii) whether the instructor possesses a master’s degree in philosophy [32]. Teachers Frans and Peter do not have a master’s degree in philosophy, but the others have; (iii) the instructor’s years of teaching experience following the obtainment of his or her degree in teaching philosophy [33]. For one teacher we have ‘0 years’, i.e. he is unqualified for teaching philosophy, twice ‘1–5 years’, twice ‘6–10 years’ and twice ‘11–15 years’. It is assumed that students of qualified philosophy teachers will do philosophy on a higher level than students of non-qualified teachers.

A student’s age is an important characteristic, and while a significant correlation was not found between the quality of doing philosophy and age in earlier research involving learners between 10 and 16 years old [12], we nevertheless assume that older students will view the world in a less egocentric manner than their younger counterparts. Accordingly, we have included tenth, eleventh, and twelfth grade pre-university and senior general higher education students in this study. We predict that eleventh and twelfth grade students (who are generally between 17 and 18 years old) will be more likely than tenth graders to reflect in a manner wherein their common reality is not only the subject of a common discourse, but also a common construction [34]. It turned out that there were three classes with tenth graders and five classes with eleventh and twelve graders. As such, higher levels of doing philosophy are anticipated among eleventh and twelfth grade students.

Teachers were given a verbal overview of the study prior to beginning the course, and permission was obtained via email to videotape one of each instructor’s standard lessons; teachers were also provided with information to assist them in preparing their recordings. Appointments were later made with the teachers to record said lessons, who then distributed consent forms to students for their parents to sign. The forms explained the study’s aim and informed parents that their child would be videotaped. In cases wherein a parent did not provide consent, the camera was positioned in a way to ensure that their child would not be recorded. The lesson environment and content were not altered in any way for the purpose of the study, and no personal details (e.g., information pertaining to physical or mental health) were collected.

### Data collection

The data sources are the philosophy lessons and the reflections by the teachers. For each lesson, data were collected through five instruments: (i) a short list with factual questions distributed to teachers prior to the class (see S1 File); (ii) classroom recordings and transcripts; (iii) classroom observations; (iv) post-lesson short lists with factual questions administered to a small group of randomly selected students concerning their learning activities (see S2 File); and (v) from recordings and transcripts of stimulated recall interviews with teachers conducted after class (see S2 File), wherein handwritten observations were obtained.

### Instruments

For each individual lesson descriptive data were summarized in a matrix. All five instruments of data collection provide information about three substantive themes, that refer to the conceptual scheme of a philosophy lesson (Fig 1). Central in this scheme is the interaction between teacher behavior and doing philosophy by the students. The design of a lesson influences this interaction. The information in a matrix with descriptive data is ordered along these three substantive themes, namely information about (a) the design of a lesson (b) teacher behavior and (c) doing philosophy by the students, in particular information about the Pearls.

We now provide an explanation of the results of the initial analyses, in particular the operationalizations used for the design of the lesson, the teacher behavior and doing philosophy by the students. These operationalizations are summarized in a coding scheme (Table 1).

**Table 1:** Coding scheme for design of a lesson, teacher behavior and doing philosophy by students.

Dimension	Category	Description
**I. Design of a lesson**		
1. Approaches to doing philosophy	1a. Connective truth finding (Ctf)	Doing philosophy wherein students search for the truth collectively through narratives and conversations (for example, exercise Socratic method falls into Ctf and Ttf)
	1b. Test-based truth finding (Ttf)	Doing philosophy in which students search for scientific truth as practiced by scientists (for example, exercise Brainstorm falls into Ttf)
	1c. Juridical debate (Jd)	Doing philosophy by debating competing or opposite claims, after which a competent “judge” reaches a verdict (for example, exercise Thought experiment falls into Jd)
**II. Teacher behavior**		
2. Philosophical teaching styles	2a. Teaching philosophy according to historical style	Students have to interpret philosophical texts (for example, reading part of text by Kant)
	2b. Teaching philosophy according to problem oriented style	Students have to solve philosophical problems (for example court-dilemma: may drugs against cancer be copied in India?)
	2c. Teaching philosophy according to person oriented style	Students are allowed to develop their own philosophy (for example, students think unprepared about positions in the mind-body discussion)
3. Dialogue	3a. Classroom talk (reciprocal arrows between teacher and one student)	Image: the teacher is akin to a parent who helps a child learn to ride a bike while holding the child’s arm (for example, in categories closed classroom talk, open classroom talk)
	3b. Philosophical discussion (reciprocal arrows between teacher and students, or between students)	Image: the instructor is similar to a parent who teaches a child how to ride a bicycle: she pushes, lets the child go, runs after him, and catches him before he falls (for example in categories of dialogue philosophical discussion)
4. Guidance	4a. Strong guidance (closed dot teacher, open for students)	Particularly the teacher determines the substantive content during the lesson
	4b. Shared guidance (closed dot teacher and students)	The teacher and the students have a common dialogue with each other, and jointly bring in the substantive content
	4c. Loose guidance (closed dots students, open dot teacher)	The teacher is mostly withdrawn and the students determine the content
**III. Doing philosophy by students**		
5. Common concept formation	5a. Deductive ladder (Method 1)	Students make *abstract* words *more concrete* (for example, happiness-> when I was 16 years old and was on holiday abroad…)
	5b. Sentence building (Method 2)	Students *think aloud* about how a concept can be *used substantively* (for example, happiness can shake your soul)
	5c. Defining (Method 3)	Students make definitions (for example, happiness is…)
	5d. Counterexamples and exploring boundaries (Method 4)	Students search for counterexamples and explore boundaries (for example, misery, joy)
6. Pearls of doing philosophy	6a. Number of pearls	Number of different moments that doing philosophy occurs in a lesson. Doing philosophy starts with a philosophical question or proposition. Then an answer follows with subsequent steps (‘because’, logic must be used, ideally there is an example, philosophical positions are defended, then criticism follows, and finally their own position is judged). Pearls were operationalized in this study as fragments of interaction comprising a significant number of utterances made by a single participant prior to being interrupted by his or her peer. A pearl ends when all participants have finished answering the philosophical question or proposition, irrespective whether all steps are made
	6b. Percentage of duration of lesson	The percentage of time that the pearls consume is equal to the time pearls take divided by the duration of the lesson (in seconds)
7. Highest level of the pearl	7a. Rationalizing (1)	First layer of pearl: when students provide a reason for an answer to a philosophical question or proposition: ‘because’ will be a logical word where rationalizing starts. But it is necessary for this layer that *logic* is used
	7b. Analyzing (2)	Second layer of pearl: conceptual analysis explains the meaning of concepts: material is divided into pieces and it is invented how the pieces are related to each other and to the overall structure. Therefore doing philosophy starts with a philosophical question or proposition, then a reason follows (‘because’), logic is used and an example follows
	7c. Testing (3)	Third layer of pearl: when students *defend* the opinions or ideas of others
	7d. Making criticism (4)	Fourth layer of pearl: when students attack the opinions or ideas of others
	7e. Reflecting (5)	Fifth layer of pearl: when students attack their *own opinions* or ideas and reflect 1) on arguments provided pro and con, 2) on the assessment framework, and 3) on their own application of this framework

### Design of a lesson

#### Approaches to doing philosophy

A short list with factual questions distributed to teachers prior to the class, a short list of factual questions for a small group of students after the lesson, and interviews are used to code approaches to doing philosophy into variables for the design of a lesson. The approaches are Juridical debate (Jd), Connective truth finding (Ctf), and Test-based truth finding (Ttf). Three aspects were investigated in relation to these approaches for each lesson: design (i.e., what teachers planned to do), execution, and learning activities. For the *design* we focus on what teachers planned to do. Before the lessons the teachers indicated in the list of questions the exercise they planned to use. Using the clustering of the 30 philosophical exercises into three approaches to doing philosophy in a lesson [5] we were able to assign the exercise to an approach. In the *execution*, the focus is on the realization of the exercise, evaluated by the teacher in the interview after the lesson. In this evaluation they could choose from a number of characteristics of the approaches that identify these approaches ([5], Table 1). In the *learning activities* we focus on the evaluations of the majority of five randomly selected students, provided after the lesson. These students could choose from a number of characteristics of the approaches that identify these approaches as well. (In the CA that will be discussed below for each lesson the patterns for design, execution and learning activities are added up).

#### Substantive philosophical domains

Teachers selected domains that closely matched the life experiences of their students, such as philosophical anthropology (PA), ethics (Eth), and social philosophy (Soc), in addition to more abstract domains, such as theory of knowledge (ToK), logic (Log), and philosophy of mind (PhM).

### Teacher behavior

We study philosophical teaching styles, the form of the dialogue and guidance in the lesson.

#### Philosophical teaching styles

Classroom observations, recorded lessons, and transcripts were used in our investigation of philosophical teaching styles. The question was how many of the philosophical teaching styles were actually combined (‘1’, ‘2’ or ‘3’). The three styles were the historical style, the problem oriented style and the style directed towards the individual.

#### Dialogue

**Fig 3:**
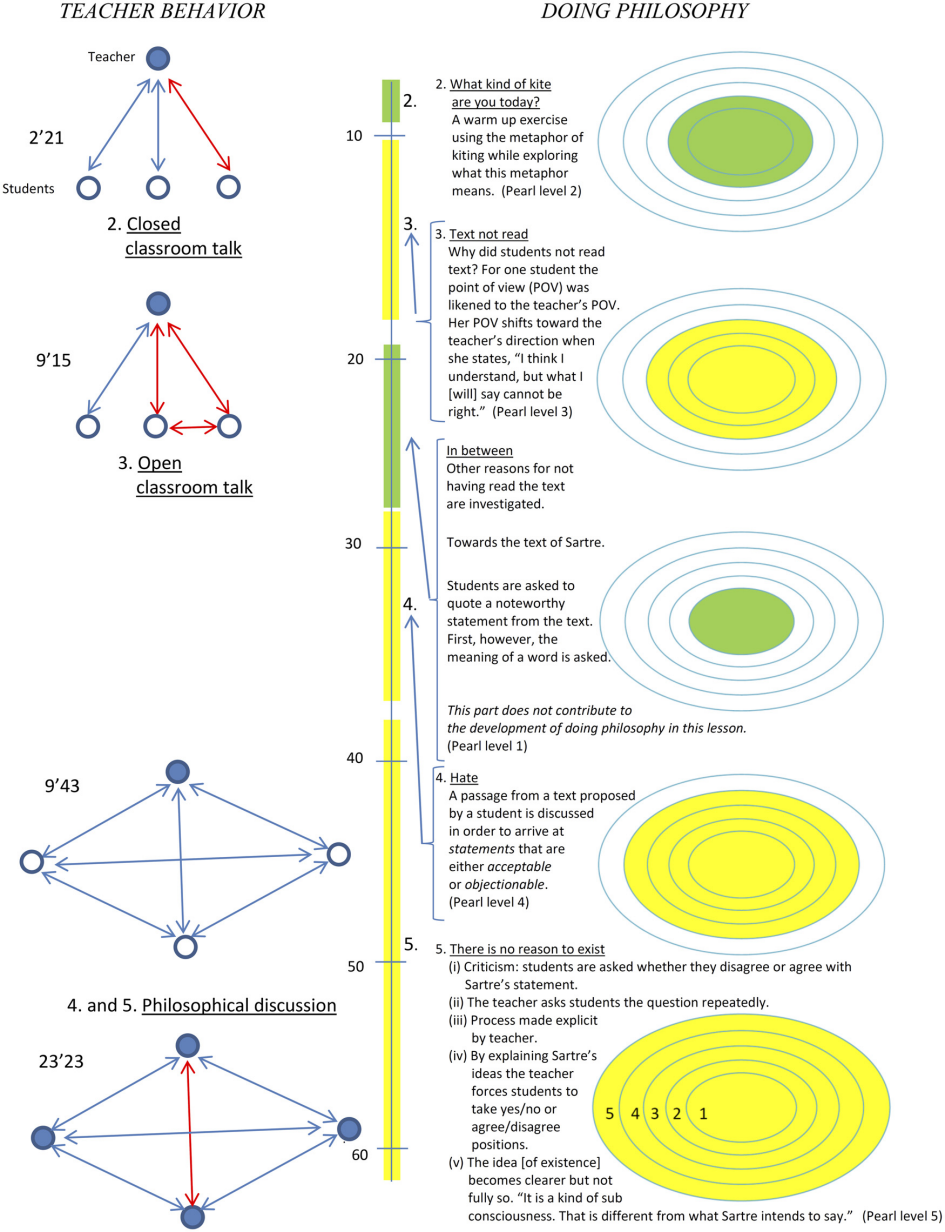
Qualitative graphical time registration of Lesson 1 taught by Oscar. The Sic et Non exercise is in yellow; other phases are in green.

The variable dialogue was coded into two categories, namely ‘philosophical discussion’ and ‘classroom talk’. A graphical time registration was used to decide whether there was a philosophical discussion or classroom talk (see Fig 3 for an example). In the graphical time registration we indicated the dialogue’s form schematically using reciprocal arrows between the teacher and his student(s), as well as between the students themselves. Arrows between a teacher and *one* student indicate classroom talk. Arrows between the teacher and students, or between students, indicate a philosophical discussion. (In the meta-matrix discussed below we denoted the most promising dialogue.)

#### Guidance

In the time registration (Fig 3) we decided on the guidance between the teacher and his students by using open or closed dots, which are connected to the arrows. *Strong* guidance by the teacher is denoted by a closed dot for the teacher and (an) open dot(s) for his student(s) respectively, while *loose* guidance is denoted by a closed and open dot for the student(s) and teacher respectively. *Shared* guidance has a closed dot for both the teacher and his student(s).

### Students doing philosophy

Based on written observations of the lessons, stimulated recall interviews were conducted with the teachers, and pearls of doing philosophy that occurred during those lessons were collectively identified. Questions were asked such as, “Do you recognize my observations [that I saw today]?,” “Do you do this more often?,” “What is your side of the story here?,” “Why did you do this, [and] why did you choose this?,” “What went well and what went wrong?,” “Where did the students make progress?,” and “Where was progress the most significant?” We study the number of pearls, the duration of the pearls (in seconds) and the percentage of the total duration in the lesson that the pearls take, the level of doing philosophy (i.e. of the pearl) reached, and common concept formation using four methods.

#### Number of pearls

The classroom observations and the interviews are used to assess the number of pearls. The eight lessons analyzed generated 31 pearls in total. Doing philosophy starts with a philosophical question or proposition. This can be a proposition taken from a philosophical text, or from a teacher, or from the students themselves. Then an answer to the question follows. When the answer is elaborate, subsequent steps are made (‘because’, logic must be used, ideally there is an example, philosophical positions are defended, then criticism follows, and finally their own position is judged). Pearls were operationalized in this study as fragments of interaction comprising a significant number of utterances made by a single participant prior to being interrupted by his or her peer. Thirty-one pearls were generated during the eight lessons, which varied in range from 3 to 41 utterances. An utterance is defined as a sequence of words by a person that ends when it is interrupted by another person. A pearl ends when all participants have finished answering the philosophical question or proposition, irrespective of whether all steps are made. After this pearl there may be a moment of instruction by the teacher, or a new question or proposition that—when the answer to the question or proposition is not ‘yes’ or ‘no’—can lead to a new pearl. Transcripts for two pearls are provided below.

#### Percentage of duration of lesson

The length of lessons is not identical in every school. We encountered lessons of 50, 60 and 70 minutes. The length of pearls is not identical as well. We found examples of pearls having a length of 75, 583, 875 and 1,403 seconds (i.e. 1’15, 9’43, 14’35 and 23’23 minutes). Video recordings, transcripts, observations, and interviews were used to assess the duration (in seconds), and the percentage of class time that the pearls consumed.

#### Highest level reached

Video recordings, transcripts, observations, and interviews were used to qualify each pearl according to the highest level of the Pearl Model reached, as discussed earlier in the study. For instance, if testing was the highest level achieved in a fragment of interaction, it was deemed a third level pearl. In the meta-matrix discussed below, we indicate the highest pearl level achieved by students for each lesson.

#### Common concept formation

The common concept formations for all pearls were assessed using the taxonomy of conceptual analysis, and pearls were qualified according to the most seemingly appropriate method. These methods included the deductive ladder (Method 1), sentence building (Method 2), defining (Method 3), and searching for counterexamples/exploring boundaries (Method 4). A pearl is coded into the method that was most relevant to characterize the pearl. For every lesson, the use of each method was quantified according to the percentage of time it was used in the pearls, adding up to 100.

### Initial data analysis phase and the study of individual lessons

For each lesson, data was examined in light of qualitative graphical time registration and by displaying it in matrices [28]. The complete materials were used to demonstrate that the concepts proposed in the study’s theoretical framework sufficiently covered the data, which is further discussed in the Data quality and reliability section.

During the first step of analysis, recordings and transcripts from two lessons were examined using thick description [35]. Lesson 1 (taught by Oscar) and Lesson 7 (taught by Frans) furnished an analysis framework. Subsequently, two parallel cases (Lessons 2 and 8 taught by Marc and Peter respectively) were analyzed. Generally, results from the second two lessons were in agreement with the first two, thereby sharpening the analysis framework.

Graphical time registration of lessons were conducted using recordings, transcripts, observations, and interviews in order to graphically summarize the relationship between students doing philosophy and teacher behavior. An illustration is provided in Fig 3. The vertical line in the middle represents the duration of the lesson. Teaching is displayed to the left of the vertical line and students doing philosophy to the right. Fig 3 shows, from left to right, (i) the duration and form of the interaction, and the contribution to the interaction; (ii) the duration of pearls and non-pearls; (iii) additional contextual notes, and (iv) the quantity of pearls, and levels. We provide a reading instruction to show what doing philosophy looks like in a lesson where the teacher uses the exercise Sic et non (of course, this is not necessarily a typical application of Sic et non). (i) On the left the length of each of the four moments of doing philosophy in classroom is provided (2’21, 9’15, 9’43 and 23’23). Twice we see classroom talk with strong guidance, once philosophical discussion with strong guidance, and once philosophical discussion with shared guidance. (ii) The lesson by Oscar took 100 minutes. Displaying approximately 50 minutes is sufficient for providing an understandable reading instruction. The displayed pearls of doing philosophy are displayed in yellow. A fraction that cannot be characterized as a contribution to doing philosophy is displayed in green. (iii) There are additional contextual notes for the pearls and the fraction that cannot be characterized as a pearl. (iv) In the complete lesson there where six pearls that took 60% of the duration of the lesson. On the right we displayed pearls 2, 3, 4 and 5. The highest level found was level 5 (reflecting).

A submatrix was used to summarize the complete data for each lesson, and the information contained within it was categorized according to the conceptual framework in Fig 1. Hence, it includes information pertaining to design of a lesson, teacher behavior, and students doing philosophy. These three parts were then examined based on the distinction between an instructor’s teaching and student learning present in output driven activities, as well as the learning processes described in the theoretical framework (i.e., design, execution, learning activities, and learning results).

### A meta matrix for comparing individual lessons

The initial data analysis outcomes for each of the eight lessons are summarized in a meta-matrix [28], which includes results for teacher behavior, variables for design of a lesson, and students doing philosophy (see Table 2). The lessons are in the columns and the variables in the rows.

**Table 2:** Meta-matrix with results for variables in the context of design of a lesson, teacher behavior, and students doing philosophy for eight philosophy lessons.

VARIABLES		Lesson 1	Lesson 2	Lesson 3	Lesson 4	Lesson 5	Lesson 6	Lesson 7	Lesson 8
		*Oscar*	*Marc*	*Marc*	*Jos*	*Ger*	*Marlies*	*Frans*	*Peter*
**I. Design of a lesson**									
*Approaches to doing philosophy*									
Connected truth finding (Ctf), Test-based truth finding (Ttf), Juridical debate (Jd)	Design	Jd	Jd	Ctf & Ttf	Ctf & Ttf	Ttf	Jd	Ctf	Ctf
	Execution	Ttf	Ttf	Ttf	Ttf	Ttf	Ctf	Ctf	Ttf
	Learning activities	Ttf	Jd	Jd	Ctf	-	Ctf	Ttf	Ctf & Ttf
*Domains*		Philosophical anthropology and logic	Philosophy of mind	Philosophical anthropology	Philosophical anthropology	Theory of knowledge	Social Philosophy	Ethics	Philosophical anthropology
*Teacher characteristics*									
Aim fulfilled		No	Yes	Yes	No	No	No	No	No
MA in philosophy		Yes	Yes	Yes	Yes	Yes	Yes	No	No
Years of experience after training (categorized)		11–15	11–15*	11–15*	1–5	1–5	6–10	0**	6–10***
Number of teaching styles		3	1	1	1	1	1	1	1
*Student characteristics*									
Grade		10	11	12	11	11	11	10	10
**II. Teacher behavior**									
Interaction	Design of exercise	Sic et Non	Thought experiment	Socratic method	Socratic method	Brainstorm	Speech	Classroom talk	Presentation
	Execution of exercise (dialogue)	Philosophical discussion with shared guidance	Philosophical discussion with shared guidance	Philosophical discussion with shared guidance	Classroom talk with loose/shared guidance	Classroom talk with shared guidance	Classroom talk with loose guidance	Classroom talk with strong guidance	Classroom talk with loose guidance
**III. Students doing philosophy**									
Number of pearls		6	3	4	5	3	5	3	2
Pearls (percentage of lesson)		60%	34%	25%	28%	23%	13%	28%	10%
Highest level reached (1–5)		5	5	5	4	4	4	4	4
Common concept formation in taxonomy of conceptual analysis ****	Method 1	39%	0%	0%	39%	0%	19%	55%	0%
	Method 2	29%	53%	13%	15%	0%	16%	26%	0%
	Method 3	0%	0%	29%	34%	13%	27%	0%	100%
	Method 4	32%	47%	58%	12%	87%	38%	19%	0%

Note. The Sic et Non exercise was used in Oscar’s philosophy lesson, which matches the criteria of the juridical debate approach. Nonetheless, the exercise’s execution and learning was more akin to the test-based truth finding approach. The substantive domain was philosophical anthropology and logic. The goal of learning philosophy by doing philosophy was unfulfilled. Oscar possesses a master’s degree in philosophy and has 11–15 years of post-training teaching experience. He used three different philosophical teaching styles, and his students were tenth graders. The dialogue was a philosophical discussion with shared guidance. Six pearls were observed that spanned 60% of the lesson’s duration, and the fifth level (reflection) was the highest reached. Of the time pearls were observed, 39%, 29%, and 32% utilized methods 1 (deductive ladder), 2 (sentence building), and 3 (counterexamples/exploring boundaries) respectively.

*Obtained a master’s degree in teaching philosophy after completing a regular curriculum in teacher training.

**Does not possess a degree in teaching philosophy.

***Possesses an academic degree in another field, but received training to teach philosophy.

****Method 1 = deductive ladder; Method 2 = sentence building; Method 3 = defining; Method 4 = counterexamples/exploring boundaries.

We provide a reading instruction for the lesson of Oscar, who is in the first column. The Sic et Non exercise was used in Oscar’s philosophy lesson, which matches the criteria of the juridical debate approach. Nonetheless, the exercise’s execution and learning activity was more akin to the test-based truth finding approach. The substantive domain was philosophical anthropology and logic. The goal of learning philosophy by doing philosophy was unfulfilled. Oscar possesses a master’s degree in philosophy and has 11–15 years of post-training teaching experience. He used three different philosophical teaching styles, and his students were tenth graders. The dialogue was a philosophical discussion with shared guidance. Six pearls were observed that spanned 60% of the lesson’s duration, and the fifth level (reflection) was the highest reached. Of the time pearls were observed, 39%, 29%, and 32% utilized methods 1 (deductive ladder), 2 (sentence building), and 3 (counterexamples/exploring boundaries) respectively.

#### Correspondence analysis of the meta-matrix

The meta-matrix was analyzed using CA [36] (IBM SPSS-routine ANACOR, syntax is in S3 File). CA is a statistical tool for descriptive analysis of data (see also S4 File).

Prior to analyzing the meta-matrix using CA, it first had to be transformed into a so-called super-indicator matrix [36], see Table 3 and S1 Table, syntax is provided in S3 File. In this super-indicator matrix the lessons were arranged in eight rows and the variable levels in columns. For example, the dialogue for teacher behavior was coded into two variables: one with philosophical discussion and classroom talk as levels, and the other with loose, shared, and strong guidance as levels.

**Table 3:** Super-indicator matrix of the meta-matrix in Table 2. For the abbreviations of the labels for the levels, see Table 2. Eight rows (lessons) and thirteen variables that have in total 37 levels.

	Approaches			Domains						Aim		MA Phil.		Exp. after training				St. grade	
	Jd	Ttf	Ctf	PA	PhM	ToK	Soc	Eth	Log	yes	no	yes	no	0	1/5	6/10	11/15	10	11/12
1.	1	2	0	.5	0	0	0	0	.5	0	1	1	0	0	0	0	1	1	0
2.	2	1	0	0	1	0	0	0	0	1	0	1	0	0	0	0	1	0	1
3.	1	1.5	.5	1	0	0	0	0	0	1	0	1	0	0	0	0	1	0	1
4.	0	1.5	1.5	1	0	0	0	0	0	0	1	1	0	0	1	0	0	0	1
5.	0	2	0	0	0	1	0	0	0	0	1	1	0	0	1	0	0	0	1
6.	1	0	2	0	0	0	1	0	0	0	1	1	0	0	0	1	0	0	1
7.	0	1	2	0	0	0	0	1	0	0	1	0	1	1	0	0	0	1	0
8.	0	1.5	1.5	1	0	0	0	0	0	0	1	0	1	0	0	1	0	1	0
*(continued)*																			
	Teaching styles		Dialogue		Guidance			# Pearls		Duration (%)			Highest level		Methods common concept formation				
	1	3	disc	crt	loo	sh	str	23	456	lo	mid	high	4	5	M1	M2	M3	M4	
1.	0	1	1	0	0	1	0	0	1	0	0	1	0	1	.39	.29	0	.32	
2.	1	0	1	0	0	1	0	1	0	0	0	1	0	1	0	.53	0	.47	
3.	1	0	1	0	0	1	0	0	1	0	1	0	0	1	0	.13	.29	.58	
4.	1	0	0	1	.5	.5	0	0	1	0	1	0	1	0	.39	.15	.34	.12	
5.	1	0	0	1	0	1	0	1	0	0	1	0	1	0	0	0	.13	.87	
6.	1	0	0	1	1	0	0	0	1	1	0	0	1	0	.19	.16	.27	.38	
7.	1	0	0	1	0	0	1	1	0	0	1	0	1	0	.55	.26	0	.19	
8.	1	0	0	1	1	0	0	1	0	1	0	0	1	0	0	0	1	0	

In the super-indicator matrix, a lesson was allotted a 1 for the levels that it fell into, and a 0 for all others. Lesson 1, for example, was given a 1 for philosophical discussion and shared guidance. If a lesson falls into two levels for the same variable, however, it receives .5 for each (fuzzy coding) [36]. Four methods were present for the common concept formation variables, which were allotted proportions that totaled 1. In full lessons, pearls were observed for a proportion of time and then coded into the super-indicator matrix in lower, middle, and higher levels (higher and lower level pearls were those with >34% and <13% occurrence rates respectively). Details are in S5 File.

In the graphical representation of lessons, rows that are close in proximity indicate similarity and the presence of many levels of common variables. Likewise, columns that are close in proximity denote the presence of similar variable levels, and thus indicate that they were used in identical lessons. Hence, the graphical representations of the lessons (rows) and levels (columns) are closely related. Apart from a scaling factor, the lessons are included in the mean of the levels used by them, and the levels are included in the mean of the lessons in which they were used. Interpretation principles for CA are presented in S4 and S5 Files.

### Data quality and reliability

In qualitative research, steps must be taken to ensure data integrity. As such, the present study implemented safeguarding procedures based upon those proposed by Denzin and Lincoln, which were developed in order to address key concerns related to data collection and analysis [35], [28], [37]. Our first precaution involved ensuring that a comfortable distance was maintained, both physically and psychologically, between the researchers and those being observed. From a physical standpoint this was achieved by using a camera mounted on a tripod while recording each lesson. The resultant recordings were then examined (i) by summarizing doing philosophy as presented in the lesson so that our interpretation could be verified and/or supplemented by the instructor during their respective interviews; (ii) by coding pearls that the teacher and observer agreed upon into a graphical time registration following data collection; and (iii) by using one part of the recordings to develop a conceptual pearl model, wherein the complete materials were used to demonstrate that the concepts proposed in the study’s theoretical framework were sufficiently covered in the data. Additionally, as data collection was conducted, a log was maintained in order to document the decision-making process.

Recordings and transcripts were evaluated by others, including the seven teachers who participated (member checks), our colleagues (peer debriefing), and expert reviewers acquainted with philosophy teaching methodologies. Colleagues assisted in examining the interpretations presented in the study’s literature review, in addition to our analyses of the results, both in cases in which we doubted our conclusions and in instances wherein our interpretations seemed relatively certain.

The interim results were also presented at a gathering of Dutch scholars in the field of philosophy teaching methodologies, who verified our interpretations and performed an independent assessment of 22 of our interpretations of the pearls. For those pearls, the average interrater reliability for the Pearl Model levels and methods of common concept formation was 60%. It should be noted, however, that the raters were previously unfamiliar with the Pearl Model and the common concept formation methods. Furthermore, they were untrained and did not exchange information amongst themselves or with the researchers.

## Results

**Fig 4:**
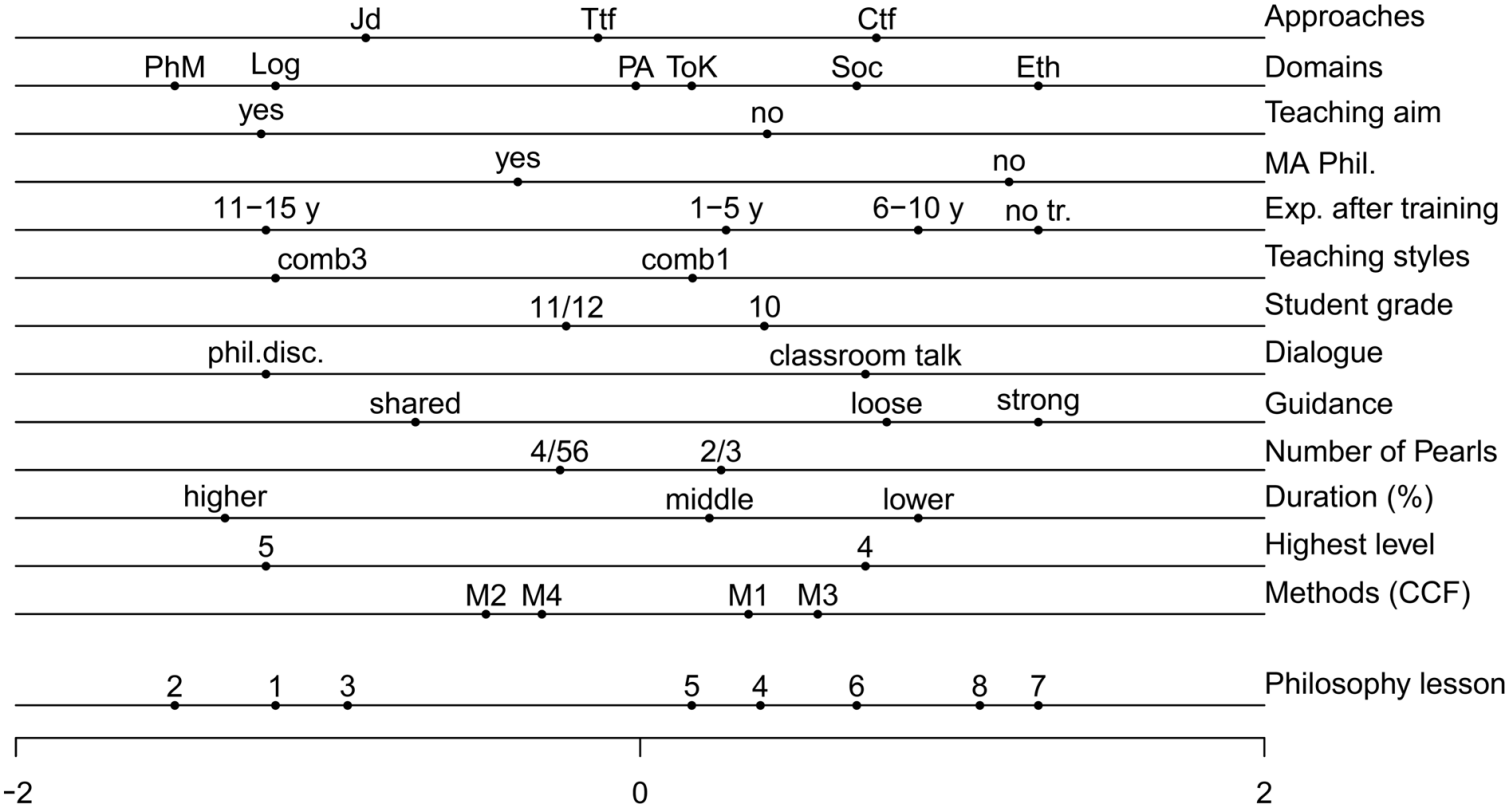
First dimension of the correspondence analysis.

The super-indicator-matrix (see Table 3) was analyzed using CA. CA of the super-indicator matrix yielded a dominant first dimension as the lessons and variable categories in the two-dimensional solution (not shown here) were in the form of a horseshoe [36]. As such, only the first dimension of the CA solution was discussed in this study. In Fig 4, each variable was been placed on a separate horizontal line, beginning with approaches and ending with methods; the dots scattered across the bottom line represent each lesson.

An examination of Fig 4 reveals that lessons 1–3 are on the left of the origin and lessons 4–8 on the right. CA indicates that doing philosophy was more effective in lessons 1–3. The lower four lines show that students doing philosophy produced a larger number of pearls (between 4 to 6), which encompass a substantial percentage of each lesson’s duration, wherein the highest level (5, reflection) was often reached. Furthermore, methods 2 (sentence building) and 4 (searching for counterexamples/exploring boundaries) were more frequently employed in these lessons, and teacher behavior often led to philosophical discussion with shared guidance.

Among the variables in the context of design of a lesson in lessons 1–3, Jd occurred most frequently in conjunction with more abstract domains, such as philosophy of mind and logic. The teachers generally aimed to teach philosophy by doing philosophy, and the majority possessed a master’s degree in philosophy, as well as many years of teaching experience after training. Of the instructors, only one was capable of using each of the three philosophical teaching styles concurrently. Students in lessons 1–3 primarily included eleventh and twelfth graders, who on average were between 17 and 18 years old.

In lessons 4–8 the number of pearls was smaller (between 2 to 3) and constituted a limited percentage of the lesson’s total duration. Generally, the second highest level (4, producing criticism) was reached in these lessons, and greater use of methods 1 (deductive ladder) and 3 (defining) was observed. Teacher behavior often led to classroom talk, with either loose or strong guidance.

As for the context variables in these lessons, doing philosophy as a form of Ctf occurred most frequently, alongside domains that closely matched the students’ life experiences, such as ethics and social philosophy. Teachers of these lessons did not aim to teach philosophy by doing philosophy. Moreover, they were relatively less likely to possess a master’s degree in philosophy, and had relatively less teaching experience after training. Students in this group included mostly tenth graders with an average age of 16.

To assess the importance of the variables it is possible to calculate their contribution to the first dimension (see S6 File, for details). The average contribution of the variables is .067 (= 1/15, where Approaches is counted thrice). The variables that contribute more than average are, from top to bottom in Fig 4, Domains (.086), Aim (.068), Experience after training (.133), Dialogue (.120), Guidance (.095), % Pearls (.091) and Highest level (.120). In particular the teaching experience after training, dialogue, guidance and the highest pearl level reached support the interpretation that the combination of teacher experience and teacher behavior (dialogue and guidance) influence whether the highest level of the pearl is reached.

As the number of cases is smaller than the number of (levels of the) variables, we studied the stability of the quantifications of the cases (lessons) on the first dimension (see also S2 Table and S7 File). For this purpose we carried out 13 additional analyses with 12 variables, leaving out each of the 13 variables one at a time. We then calculated the correlation between the original quantifications of the lessons (lowest line in Fig 4) and the quantifications of each of the 13 additional analyses. The correlations ranged from .992 to .999. We also studied the impact of leaving out the three variables that contribute most to the first dimension, Experience after training, Dialogue and Highest level. For the remaining 10 variables the correlation still is .955. A last way to study the stability is by leaving out cases. We left out pairs of cases that are likely to be most influential (compare Fig 4): cases 2 and 7, cases 1 and 2 and cases 7 and 8. If we leave out these pairs and calculate the correlation with the quantifications of the remaining 6 lessons in the full solution we find correlations of, respectively, .990, .988 and .981. This shows that the quantifications of the lessons, that we interpret as a dimension of effectiveness of doing philosophy, is very stable. As the lessons are included in the mean of the levels used by them (see Methods section), this leads to the conclusion that overall the quantification of the levels of the variables is rather stable.

We illustrate CA with two examples, namely the two most extreme lessons. The most effective lesson was Lesson 2 taught by Marc. A segment from this lesson is described below.

### An effective lesson: Lesson 2 taught by Marc (Pearl 3)

#### Context

This is a 50-minute lesson from an eleventh grade class comprising 13 female students, wherein three pearls have been identified. Tables in the classroom were arranged in a U-shape, and learners were commencing a new module entitled Philosophy of Mind. Marc began with an overview of the reading materials, and discussed the mind while prompting students to contemplate what it is and how it functions. He then talked about Alan Turing and the Turing Test prior to asking students to formulate questions that might help one to distinguish between a human and a computer. After Marc responded to some inquiries concerning the task, students began writing their questions (Pearl 1, 3 minutes). Next, Marc conducted a philosophical discussion with students concerning how a human might be asked a question three different times (Pearl 2, 8 minutes). Afterwards, a philosophical discussion took place (Pearl 3, 8 minutes). An excerpt from Pearl 3 follows.

#### Thought experiment

**Student 1:** Yes, if you ask a computer [to] “describe me as… as you see me now,” then the computer has to judge you at that moment

**Marc:** Yes, but in a figurative sense, right, because you do not have a camera or so

**Student 1:** Yes, yes, as a person

**Marc:** And then he should say something like “quite OK”, something like that?

**Student 1:** Yes

**Marc:** Would that work?

**Student 2:** … quite OK, you cannot… you are quite OK... [inaudible]

**Student 1:** No, but you cannot…[inaudible]

**Marc:** And why do you consider this to be a good question?

**Student 1:** Because a human has knowledge of human nature and a computer does not.

**Marc:** So the rendering of answers that you give concerning what kind of person you are—that is something a human is capable of, to a certain extent, but for a computer—yes, this question is way too difficult because [the computer] does not have knowledge of human nature, right? Is this a good idea?

**Student 3:** Yes, it depends, because there are also many of these stupid people and then you tell them, yes, what do you actually know about me, “quite OK”? I think that a computer will also be able to do this so this does not reveal whether it is a computer or a person

**Marc:** Some people are not very capable to do this, right

**Student 3:** No, there are enough people who have something like: yes, you are quite OK

**Student 1:** In fact you always have to assume that people are always stupid, then it is easy, you can just say that a computer is a person who is not so smart

**Student 3:** But I thought the point is that a person... [inaudible]

[talking at the same time]

**Student 1:** Yes, but...

[talking at the same time]

**Student 1:** But if you ask smart questions, and the computer gives great answers, because it is just like a person and that he then says, concisely, if you ask me, describe me in a few words

**Student 4:** But you have such people, people that are super smart but who are socially nobodies

**Student 1:** Yes but, no—ok, if you ask me to describe myself in three words, then I always get responses such as noisy or amusing, and a computer cannot provide such answers…and if you are talking all the time a computer cannot say “be quiet.”

**Marc:** Ok, but this does not necessarily mean that you are dealing with a stupid person since you received all kinds of smart answers, so it is possible that he considers this to be a hard question. A human can have this opinion, but a computer can also have this opinion; this does not help you to make a distinction, but your point is simply that a computer will not be able to do this because it does not have knowledge of human nature.

**Student 1:** Yes.

**Marc:** Yes…

**Student 5:** But see, this human and this computer only know you objectively. They do not know you as a friend or as your parents know you. I would also not be able to say what kind of person this is if someone would ask me such a question.

#### Analysis

Reflection takes place in this pearl, since the student thinks on a high level about earlier contributions to the discussion. First, the idea that there is no distinction between a human and a computer is disputed. Next, a distinction is made between objects and subjects, wherein the student defines herself as an object (such as a computer) in a scenario in which an unknown person asks her something. Thus, the student questions her earlier opinion, since she initially made a distinction between herself and a computer. Furthermore, the students respond in an authentic manner by characterizing their peer as noisy or amusing, and Marc structures the discussion in a way that encourages reactions between students.

### A less effective lesson: Lesson 7 taught by Frans (Pearl 1)

The less effective lesson was lesson 7 by Frans. We also provide a part of the lesson.

#### Context

The following is a 50-minute lesson from a tenth grade class comprising 15 students, of which 12 were female and 3 male. There were fifteen tables in the classroom arranged three rows of five. The course’s textbook was entitled *Happiness and Wisdom for Beginners*, and students had reached its ninth chapter, which focused on participation. Three pearls were identified in this lesson. The first pearl emerges when Frans commences the lesson by asking learners to consider whether student/teacher participation exists at their school; afterwards, 14 minutes of classroom talk transpires, which comprises part of this pearl. In the excerpt below, Frans engages his students in a series of questions and answers.

#### Classroom talk

**Frans:** It [the question] is about participation. I was asking myself whether there is student participation at this school.

…

**Student 1:** There is not much participation at this school, simply because we are not allowed to go on strike tomorrow.

**Frans:** And why are you not allowed to go on strike?

**Student 1:** Because the school is not democratic enough.

**Frans:** We are simply not democratic enough?

**Student 1:** Not directly, but indirectly.

**Frans:** Ah, so you would like to go on strike?

**Student 1:** No, not really.

…

**Student 2:** Why will you not go on strike?

**Frans:** Tomorrow is the day students will go on strike, as organized by the student union.

**Student 3:** Under the guidance of teachers.

**Frans:** Under the guidance of teachers?

**Student 4:** Go for it!

**Frans:** Yes, in the near future it will be proposed that we have a six-week summer holiday instead of the usual seven, but that is [only] for teachers, and they will be able to schedule these days differently for this [upcoming] school holiday. The school administration could say [that they] will do this at other times [as well], for example by allotting a few more days off in May [and by] arranging things in slightly different ways. But the holidays for teachers will be one week shorter. This will not be discussed with us. Actually, [the school administration] discusses less and less with us [nowadays]. That is kind of sad. Although we have representatives on the school board, we rarely correspond with them, which [again] is kind of sad. Nevertheless, philosophers have contemplated [the concept of] participation. How might you organize [participation] in an optimal way?

#### Analysis

It can be deduced that testing takes place in this pearl, since the concept of participation is questioned based on a personal experience. Moreover, there is limited substantial mutual input in this interaction. For example, when the student alludes to the distinction between direct and indirect democracy, the teacher instead shifts the conversation in a different direction.

## Discussion

The aim of this study was to gain greater insight into doing philosophy effectively in classroom teaching. In our observational study we find that doing philosophy effectively goes together with shared guidance as well as with the approach Jd. As this is an observational study, we cannot conclude on effects. However, there is an indication that shared guidance rather than the approach to which a philosophical exercise belongs primarily predicts doing philosophy effectively. Moreover, there is no indication that a direct relationship exists between specific exercises and doing philosophy effectively, which is underpinned by Lesson 6. In Lesson 6 the exercise was speech, a Jd approach that should in principal promote doing philosophy effectively. However, the students worked independently during the lesson with loose guidance by the instructor, and merely partook in classroom talk rather than a philosophical discussion. This suggests that when it comes to doing philosophy effectively, guidance is the determining factor and not the approach of the exercise. The CA further supports this interpretation, wherein guidance and approach respectively contributed 1.5 and .75 times the average contribution of a variable to the CA solution. Additionally, doing philosophy effectively was more closely related to Jd than Ctf. This is an important and surprising finding since Ctf is a dominant approach in Dutch secondary school philosophy classrooms [1]. Further study is required in order to determine how this relates to the importance of guidance (i.e., how shared guidance in Ctf leads to doing philosophy more effectively).

The results raise a number of questions. First, is it possible that the application of one exercise more naturally leads to a specific approach than another? For example, Sic et Non seems to lead automatically to Jd, although the Socratic method relates to both Ctf and Ttf. Second, are the Ctf and Ttf approaches dominant in Dutch secondary school philosophy classrooms? Furthermore, does the use of exercises meeting the Jd approach’s criteria benefit learning? Indeed, doing philosophy effectively appears to be more easily accomplished by employing exercises from certain approaches. If this is true, could exercises be developed to increase the effectiveness of less effective approaches?

Finally, it is preferable for a lesson to include (i) many pearls, (ii) lengthy pearls, and (iii) high level pearls. However, while the teachers of more effective lessons taught relatively few classes on the day that they were observed, they were nevertheless exhausted by the lesson’s end. As such, a full school day of effective lessons may not be feasible. It is also possible that teachers give a mixture of effective and less effective lessons. Recent research (for example Lesson Study) suggests that teachers design better lessons when they work under academic guidance and that their students achieve better learning results [38–40]. This may be a topic for further study.

## Conclusions

This study examined the relationship between teacher behavior and how students do philosophy, while also attempting to determine what makes doing philosophy effective. In pursuing this goal, we sought specifically to ascertain (i) which teaching behaviors and student learning activities would be observed during a philosophy lesson and how they would interact; (ii) the shape that philosophical approaches would take during interactions facilitated by the philosophical exercises; and (iii) the forms of interaction that would be observed in relation to the substantive philosophical domains.

The above questions were key in formulating a number of research expectations. Before proceeding to summarize the conclusions, however, it is important to note that they are based on only eight lessons, and therefore one should be cautious in generalizing the study’s findings. Our first expectation was that higher levels of doing philosophy go together with more reflexive forms of common concept formation. Indeed, CA revealed that lessons embodying higher levels of doing philosophy (e.g., lessons 1–3) employed relatively greater use of sentence building and searching for counterexamples/exploring boundaries (see Fig 4), thus confirming this expectation and also part of the first research question concerning student learning activities.

Our second expectation was that, when compared with classroom talk, higher levels of doing philosophy would be encountered in philosophical discussions. The third expectation was that shared rather than loose or strong guidance would more often produce high levels of doing philosophy, an outcome that was apparent in more effective lessons. The fourth expectation was that doing philosophy effectively will occur more easily when a teacher is able to combine more than one philosophical teaching style in a lesson. Thus, both the second and third research expectations were confirmed, and consequently part of the first research question concerning teaching behaviors and their interactions. There was limited (positive) evidence for the fourth expectation as only one teacher used three teaching styles whereas the other teachers used only one style.

The fifth expectation was that Ctf would occur less frequently in higher levels of doing philosophy. As predicted, Ctf exhibited greater frequency on average in lessons with relatively low levels of doing philosophy. Furthermore, Jd occurred more often in effective lessons, although Ttf existed in both effective and less effective lessons. Hence, the fourth research expectation and second research question were confirmed.

Our sixth expectation was that it would be possible to do philosophy on a high level regardless of domain. However, abstract domains were usually characteristic of effective lessons, while domains closely matching students’ life experiences were typical of less effective lessons. As such, this expectation could not be substantiated, although it did sufficiently answer the third research question.

The seventh expectation was that students of qualified teachers would do philosophy on a higher level than students of unqualified teachers. This was indeed confirmed. Finally, the eighth expectation was that eleventh and twelfth grade students would achieve higher levels of doing philosophy. This assumption was not adequately confirmed.

From a methodological perspective, this study showed how to score the effectivity of doing philosophy in lessons. The Pearl Model, Graphical time registration and the First dimension of the CA are also suitable for observing lessons in practice, and are currently used by us for coaching and training philosophy teachers, and in philosophy textbooks.

## Supporting Information

S1 File Short list with factual questions distributed to teachers prior to the lesson. (DOCX)

S2 File Post-lesson list with factual questions both for students and for teachers (as part of interview). (DOCX)

S3 File Syntax file for analyzing super-indicator matrix as well as carrying out stability analyses. (DOCX)

S4 File Correspondence analysis (CA), interpretation. (DOCX)

S5 File CA, super-indicator matrix and Burt matrix. (DOCX)

S6 File CA, contributions of variables. (DOCX)

S7 File CA, stability. (DOCX)

S1 Table Super-indicator matrix, .sav file for SPSS. (SAV)

S2 Table Data for stability analyses, .sav file for SPSS. (SAV)

## References

[1] Marsman P (2010) Vakdossier filosofie. Enschede: Stichting leerplanontwikkeling (SLO).

[2] Duffy TM, Cunningham DJ (1996) Constructivism: Implications for the design and delivery of instruction In: Jonassen DH Handbook of research for educational communications and technology. London: Prentice Hall International.

[3] Glaser R (1991) The maturing of the relationship between the science of learning and cognition and educational practice. Learning and Instruction 1, 129–144.

[4] Karskens M (2006) Waarheid als Pluraliteit en Praktijk. Filosofie en Praktijk 27, 2:10–24.

[5] Kienstra N, Karskens M, Imants J (2014a) Three approaches to doing philosophy: A proposal for grouping philosophical exercises in classroom teaching. Metaphilosopy 45, 2:288–319.

[6] Kienstra N, Karskens M, Imants J (2014b) Filosoferen in de klas: een analyse van filosofische werkvormen / Doing philosophy in classroom teaching. Tydskrif vir Geesteswetenskappe 54, 4:787–805.

[7] Fullan M (2007) The six secrets of change. San Francisco: Jossey-Bass.

[8] Thijs A, Van den Akker J (2009) Leerplan in ontwikkeling. Enschede: Stichting leerplanontwikkeling (SLO).

[9] Van den Akker JJH (2003) Curriculum perspectives: An introduction In: Van den Akker J, Kuiper W, Hameyer U, editors. Curriculum landscape and trends. Dordrecht: Kluwer.

[10] Imants J (2010) Beter leren door leiderschap. Hengelo: Hogeschool Edith Stein/ Onderwijskundigcentrum Twente en Expertis.

[11] Karskens M, Van Haperen T, Hogenbirk J, Schwab H, Wessels H (2008) Filosofie VWO Syllabus centraal examen met ingang van 2010. Utrecht: Centrale Examencommissie Vaststelling Opgaven Vwo, Havo, Vmbo.

[12] Rondhuis T (2005) Philosophical talent: Empirical investigations into philosophical features of adolescents’ discourse. Rotterdam: Veenman.

[13] St. Edward’s University Center for Teaching Excellence (2004) Bloom’s task-oriented question construction wheel (Polygon). Available: http://think.stedwards.edu/cte/content/resources. Accessed 23 December 2014.

[14] Vermunt J (1998) Het leren van leerlingen In: Verschaffel L, Vermunt J, editors. Onderwijskundig lexicon editie III. Alphen aan den Rijn: Samsom Tjeenk Willink.

[15] Imants J, Oolbekkink H, editors (2009) Leren denken in de schoolvakken. Antwerpen: Garant.

[16] Van de Ven P, Martens J, Imants J (2005) Praktijkgericht onderzoek bij de ontwikkeling van actief en zelfstandig leren binnen het schoolvak Nederlands. Pedagogische studiën 82, 293–309.

[17] Martens E, Van der Aart I (2000) Spelen Met Denken over filosoferen met kinderen. Rotterdam: Lemniscaat.

[18] Knezic D, Wubbels T, Elbers E, Hajer M (2010) The Socratic dialogue in teacher education. Teaching and Teacher Education 26, 1104–1111.

[19] Brüning B (2003) Philosophieren in der Sekundarstufe Methoden und Medien. Weinheim, Basel, Berlin: Beltz Verlag.

[20] Perkins DN (1992) Smart Schools, Better Thinking and Learning for Every Child. New York: The Free Press.

[21] Oolbekkink-Marchand HW, Van Driel J, Verloop N (2007) Een vergelijking van de perspectieven van docenten in het voortgezet en wetenschappelijk onderwijs op onderwijzen en leren in de context van onderwijsvernieuwingen. Pedagogische Studiën 84, 4:293–308.

[22] Young RE (1989) A critical theory of education: Habermas and our children’s future. London: Harvester Wheatsheaf.

[23] Van der Leeuw K, Mostert P (1991) Filosofie-opvatting en onderwijsstijl. VIC Tijdschrift voor Filosofieonderwijs 20, 1:15–24.

[24] Oakeshott M (1975) On human conduct. Oxford: Clarendon Press.

[25] CvE (College voor Examens) (2013) Syllabus filosofie vwo 2015. Available: https://www.examenblad.nl/examenstof/syllabus-2015-filosofie-vwo/2015/vwo/f=/filosofie_vwo_2015_def.pdf. Accessed 23 December 2014.

[26] CvE (College voor Examens) (2014) Syllabus filosofie havo 2015. Available: https://www.examenblad.nl/examenstof/syllabus-2015-filosofie-havo-nader/2015/havo/f=/filosofie_havo_2015_blauwe_vlek.pdf. Accessed 23 December 2014.

[27] Yin RK (2014) Case study research: Design and methods. Thousand Oaks, CA: Sage.

[28] Miles MB, Huberman AM (1994) Qualitative data analysis: An expanded sourcebook. Thousand Oaks: SAGE Publications.

[29] Greenacre M (2007) Correspondence Analysis in Practice. Boca Raton, CRC, second edition.

[30] Kessels J (1989) Kennis van kennis Een ontwikkelingsonderzoek in didactiek van filosofie. Utrecht: Innovatiefonds van de Rijksuniversiteit Utrecht.

[31] Van der Leeuw K, Mostert P (1988) Philosophieren lehren. Delft: Eburon.

[32] BCFVO (Begeleidingscommissie Filosofie in het Voortgezet Onderwijs) (2014) De commissie. Available: http://www.bcfvo.nl/bevoegdheid/. Accessed 23 December 2014.

[33] McCall CC (2009) Transforming thinking: Philosophical inquiry in the primary and secondary classroom. London/New York: Routledge.

[34] Van der Leeuw K (2009) Philosophy for children as educational reform In: Marsal E, Dobashi T, Weber B, editors. Children philosophize worldwide: Theoretical and practical concepts. New York: Peter Lang pp. 117–126.

[35] Denzin NK, Lincoln YS (2003) Introduction: The discipline and practice of qualitative research In: Denzin NK, Lincoln YS, editors. Strategies of qualitative inquiry (2nd ed). Thousand Oaks, CA: Sage pp. 1–45.

[36] Gifi A (1990) Nonlinear multivariate analysis. New York: John Wiley & Sons.

[37] Plomp T, Nieveen N (2009) An introduction to educational design research. Enschede: Stichting leerplanontwikkeling (SLO).

[38] Coenders F, Terlouw C, Pieters J, Dijkstra S (2010) The Effects of the Design and Development of a Chemistry Curriculum Reform on Teachers’ Professional Growth: A Case Study. Journal of Science Teacher Education 21, 5: 535–57.

[39] Fernandez ML (2010) Investigating how and what prospective teachers learn through microteaching lesson study. Teaching and Teacher Education 26, 2: 351–62.

[40] Cerbin W, Kopp B (2006) Lesson Study as a Model for Building Pedagogical Knowledge and Improving Teaching. International Journal of Teaching and Learning in Higher Education 18, 3: 250–7.

