# Supplementary material for: Doing Philosophy Effectively: Student Learning in Classroom Teaching
Source: PLoS One. 2015 Sep 17;10(9):e0137590. doi: 10.1371/journal.pone.0137590 (PMC4574705; doi:10.1371/journal.pone.0137590)
Supplement: S1 File — (DOCX) [file pone.0137590.s001.docx]

**Supporting Information**

**S1 File**

**Short list with factual questions distributed to teachers prior to the class**

How many years of teaching experience do you have in secondary schools (including this year)?

1. 0 years
2. 1 – 5 years
3. 6 – 10 years
4. 11 – 15 years
5. More than 15 years

Did you obtain a master of education in philosophy?

1. A master degree in philosophy followed by a regular curriculum in teacher training
2. Not a master degree in philosophy followed by a training to teach philosophy
3. No training
4. A master of education in another subject,

namely ……………………………………………….

1. A higher vocational teacher training,

namely ……………………………………………….

We would like to have insight into the practice of teaching the subject philosophy in secondary education. Do you practice “learning philosophy by doing philosohy”?

1. Yes, because ................................................................
2. No, because .................................................................

What is the student grade for the lesson that we are going to observe?

Yes No

Senior general higher education, level 4

Pre-university education, level 4

Senior general higher education, level 5

Pre-university education, level 5

Pre-university education, level 6

In a survey of the literature we found a large number of philosophical exercises [1]. Please clearly underline the exercises that you plan to use in the lesson that we are going to observe.

1 Classroom talk

2 Guided Socratic Discussion (GSD)

3 Night of philosophy

4 Philosophical café

5 Philosophy with children

6 Role-playing

7 SAPERE

8 Tetralogue

9 Thinking Skills (e.g., Images to Remember, Mystery, Missing Word, Contest of Values,

Semantic differential, Scenario thinking, Forbidden Word/Taboo, Odd One Out)

10 Written dialogue

11 Brainstorming

12 Case Method, Paradox, Dilemmas, and Counterexample

13 Community of Philosophical Inquiry (CoPI)

14 Conceptual analysis

15 Didactic puzzle (e.g., Deduction, Induction, Scrabble as propositional logic,

Koningsveld’s game)

16 Philosophical thinking out loud

17 Imagining thoughts

18 Language games

19 Metaphors

20 (Philosophical) reading of primary texts

21 Socratic method

22 Writing an explicatory philosophical essay

23 Dialogues of Oscar Brenifier

24 Philosophical debate and Sic et non

25 Speech

26 Symposium

27 Thought experiment

28 Critical Thinking

29 Writing an argumentative philosophical essay

30 Studium Generale
