# Supplementary material for: Doing Philosophy Effectively: Student Learning in Classroom Teaching
Source: PLoS One. 2015 Sep 17;10(9):e0137590. doi: 10.1371/journal.pone.0137590 (PMC4574705; doi:10.1371/journal.pone.0137590)
Supplement: S2 File — (DOCX) [file pone.0137590.s002.docx]

**Supporting Information**

**S2 File**

**Short list with factual questions administered after class to a small group of randomly selected students and to their teacher conducted**

Please indicate the column that best suits the exercise that was used today in the lesson ([1], Table 1):

| *1 Concepts* | Own thoughts as a result of  narrative | Every thought as a result of  texts, images, music, etc. | Transcendental / logical  assessment framework;  no fixed reference point  of experience: Kant  (*Critique of Pure Reason*) |
| --- | --- | --- | --- |
| *2 Method* | Spinning or weaving a tale | Selecting and testing of  relevant data; internal  criticism examining the  consistency of  reasoning/theory | Reflection on data; external  criticism: explicitly  saying no |
| *3 Structure* | We-narratives about origin  (*historical narratives)* | Logical structure | Argumentative structure |
| *4 Thoughts* | At metalevel: keeping sight  of the involvement of the  participants and on the  forming subgroups and  their (social-narrative)  connections amongst  themselves | Continuous metacognitive  thinking as an integral  part of the process | Plan of realistic contrasts  (repugnance) at  metalevel of criticism  (see [2], p.196–98) |
| *5 Epistemology* | Truth is a continuous,  successful, active  manipulation of the  world made by humans  (constructivism) | Truth is in all humans  (rational); obtaining  knowledge from the  outside world | The burden of proof of a  party is not sufficient to  fulfill the claim (see [2], p.197) |
| *6 Focus on* | Construction of narrative  by students | Inquiry and experiment | Debate |
| *7 Surroundings* | Politics in social interaction | Science | Administer justice |
| *8 Teacher* | Explains philosophy and  logic in narratives;  teacher’s manual with  assignments and games  guarantee the  philosophical level | Academic and trained  philosopher presiding  over the level of arguing  and testing | Academic and trained  philosopher separating  the rhetorical and quality  levels of the arguments  and helping to achieve a  final judgment |
| *9 Practice* | Democratic surrounding:  each student is  encouraged to talk and  has an equal opportunity  to contribute to the  discussion (see [3], p.105) | Striving for confirmation  (consensus) and negative  judgments (see [2], p.198) | Appeal to third body that  passes the final judgment  (with Kant, reason can  pass judgment) |
| *10 Analyzing* | Questioning; wondering | Continuous questioning;  interrogation | Problematizing; considering |
| *11 Testing* | Evaluation | Making definitions and  distinctions | Making judgments |
| *12 Producing*  *Criticism* | Reasoning; being led by  explanation/  reason/connection | Argumentation (pro and  con); constructing and  maintaining a logical  argument | Debating |
| *13 Reflecting* | Making metaremarks;  mirroring | Making creative leaps;  thinking about the  thought process itself | Reflecting (1) on the pro  and con arguments, (2)  on the assessment  framework, and (3) on  its own application |

Which three rows had the largest impact on the choice you made? Please clarify.

1. because

2. because

3. because

4. because

5. because

6. because

7. because

8. because

9. because

10. because

11. because

12. because

13. because

**References**

1. Kienstra N, Karskens M, Imants J (2014a) Three approaches to doing philosophy: A proposal for grouping philosophical exercises in classroom teaching. Metaphilosopy 45, 2:288-319.
2. Van der Kuijlen WJF (2009) An Unused but Highly Needful Concept: The Notion of Realrepugnanz in Kant’s Early Philosophy and Kritik der reinen Vernunft. Enschede: Ipskamp.
3. McCall CC (2009) Transforming thinking: Philosophical inquiry in the primary and secondary classroom. London/New York: Routledge.
