# Supplementary material for: Doing Philosophy Effectively: Student Learning in Classroom Teaching
Source: PLoS One. 2015 Sep 17;10(9):e0137590. doi: 10.1371/journal.pone.0137590 (PMC4574705; doi:10.1371/journal.pone.0137590)
Supplement: S3 File — (DOCX) [file pone.0137590.s003.docx]

**Supporting Information**

**S4 File**

**Spss syntax file for analyzing super-indicator matrix as well as carrying out stability analyses**

Cd ‘… name folder where file Kienstra et al.sav is located…’

get file ' Kienstra et al.sav '.

* CORRESPONDENCE ANALYSIS OF SUPER INDICATOR MATRIX.

anacor table=all(8,37)

/NORMALIZATION =CPRINCIPAL

/VARIANCES SINGULAR ROWS COLUMNS

/PLOT ROWS(1) COLOMNS(1) TRROWS(1) TRCOLUMNS(1) JOINT

/MATRIX OUT = SCORE('SCORE1.sav').

* STABILITY ANALYSIS, EACH VARIABLE IS LEFT OUT IN TURN.

* analysis data matrix min var 1 in meta matrix.

get file ' Kienstra et al.sav ' /drop pa to log.

anacor table=all(8,31)

/NORMALIZATION =CPRINCIPAL

/VARIANCES SINGULAR ROWS COLUMNS

/PLOT ROWS(1) COLOMNS(1) TRROWS(1) TRCOLUMNS(1) JOINT

/MATRIX OUT = SCORE('SCORE1min1.sav').

* analysis data matrix min var 2 in meta matrix.

get file ' Kienstra et al.sav ' /drop disc to crt.

anacor table=all(8,35)

/NORMALIZATION =CPRINCIPAL

/VARIANCES SINGULAR ROWS COLUMNS

/PLOT ROWS(1) COLOMNS(1) TRROWS(1) TRCOLUMNS(1) JOINT

/MATRIX OUT = SCORE('SCORE1min2.sav').

* analysis data matrix min var 3 in meta matrix.

get file ' Kienstra et al.sav ' /drop sh to str.

anacor table=all(8,34)

/NORMALIZATION =CPRINCIPAL

/VARIANCES SINGULAR ROWS COLUMNS

/PLOT ROWS(1) COLOMNS(1) TRROWS(1) TRCOLUMNS(1) JOINT

/MATRIX OUT = SCORE('SCORE1min3.sav').

* analysis data matrix min var 4 in meta matrix.

get file ' Kienstra et al.sav ' /drop aimyes to aimno.

anacor table=all(8,35)

/NORMALIZATION =CPRINCIPAL

/VARIANCES SINGULAR ROWS COLUMNS

/PLOT ROWS(1) COLOMNS(1) TRROWS(1) TRCOLUMNS(1) JOINT

/MATRIX OUT = SCORE('SCORE1min4.sav').

* analysis data matrix min var 5 in meta matrix.

get file ' Kienstra et al.sav ' /drop ts3 to ts1.

anacor table=all(8,35)

/NORMALIZATION =CPRINCIPAL

/VARIANCES SINGULAR ROWS COLUMNS

/PLOT ROWS(1) COLOMNS(1) TRROWS(1) TRCOLUMNS(1) JOINT

/MATRIX OUT = SCORE('SCORE1min5.sav').

* analysis data matrix min var 6 in meta matrix.

get file ' Kienstra et al.sav ' /drop maphy to maphn.

anacor table=all(8,35)

/NORMALIZATION =CPRINCIPAL

/VARIANCES SINGULAR ROWS COLUMNS

/PLOT ROWS(1) COLOMNS(1) TRROWS(1) TRCOLUMNS(1) JOINT

/MATRIX OUT = SCORE('SCORE1min6.sav').

* analysis data matrix min var 7 in meta matrix.

get file ' Kienstra et al.sav ' /drop eaT0 to eaT1115.

anacor table=all(8,33)

/NORMALIZATION =CPRINCIPAL

/VARIANCES SINGULAR ROWS COLUMNS

/PLOT ROWS(1) COLOMNS(1) TRROWS(1) TRCOLUMNS(1) JOINT

/MATRIX OUT = SCORE('SCORE1min7.sav').

* analysis data matrix min var 8 in meta matrix.

get file ' Kienstra et al.sav ' /drop stgr10 to stgr1112.

anacor table=all(8,35)

/NORMALIZATION =CPRINCIPAL

/VARIANCES SINGULAR ROWS COLUMNS

/PLOT ROWS(1) COLOMNS(1) TRROWS(1) TRCOLUMNS(1) JOINT

/MATRIX OUT = SCORE('SCORE1min8.sav').

* analysis data matrix min var 9 in meta matrix.

get file ' Kienstra et al.sav ' /drop jd to ctf.

anacor table=all(8,34)

/NORMALIZATION =CPRINCIPAL

/VARIANCES SINGULAR ROWS COLUMNS

/PLOT ROWS(1) COLOMNS(1) TRROWS(1) TRCOLUMNS(1) JOINT

/MATRIX OUT = SCORE('SCORE1min9.sav').

* analysis data matrix min var 10 in meta matrix.

get file ' Kienstra et al.sav ' /drop pearl23 to pearl456.

anacor table=all(8,35)

/NORMALIZATION =CPRINCIPAL

/VARIANCES SINGULAR ROWS COLUMNS

/PLOT ROWS(1) COLOMNS(1) TRROWS(1) TRCOLUMNS(1) JOINT

/MATRIX OUT = SCORE('SCORE1min10.sav').

* analysis data matrix min var 11 in meta matrix.

get file ' Kienstra et al.sav ' /drop durlo to durhigh.

anacor table=all(8,34)

/NORMALIZATION =CPRINCIPAL

/VARIANCES SINGULAR ROWS COLUMNS

/PLOT ROWS(1) COLOMNS(1) TRROWS(1) TRCOLUMNS(1) JOINT

/MATRIX OUT = SCORE('SCORE1min11.sav').

* analysis data matrix min var 12 in meta matrix.

get file ' Kienstra et al.sav ' /drop hile4 to hile5.

anacor table=all(8,35)

/NORMALIZATION =CPRINCIPAL

/VARIANCES SINGULAR ROWS COLUMNS

/PLOT ROWS(1) COLOMNS(1) TRROWS(1) TRCOLUMNS(1) JOINT

/MATRIX OUT = SCORE('SCORE1min12.sav').

* analysis data matrix min var 13 in meta matrix.

get file ' Kienstra et al.sav ' /drop me1 to me4.

anacor table=all(8,33)

/NORMALIZATION =CPRINCIPAL

/VARIANCES SINGULAR ROWS COLUMNS

/PLOT ROWS(1) COLOMNS(1) TRROWS(1) TRCOLUMNS(1) JOINT

/MATRIX OUT = SCORE('SCORE1min13.sav').

* analysis data matrix min the three variables that contribute most, namely Teacher exp after training, dialogue and highest level.

get file ' Kienstra et al.sav ' /drop eaT0 to eaT1115, disc to crt, hile4 to hile5.

anacor table=all(8,29)

/NORMALIZATION =CPRINCIPAL

/VARIANCES SINGULAR ROWS COLUMNS

/PLOT ROWS(1) COLOMNS(1) TRROWS(1) TRCOLUMNS(1) JOINT

/MATRIX OUT = SCORE(‘SCORE1minus3var.sav').

* … AND COLLECT ALL ROW SCORES FOR FIRST DIMENSION IN FILE. HERE THESE ARE COLLECTED IN FILE STABILITY.SAV. THEN CALCULATE CORRELATION BETWEEN FIRST DIMENSION OF SOLUTION OF ALL VARIABLES AND SOLUTIONS WHERE ONE OF MORE VARIABELE ARE LEFT OUT.

* … AND DO THREE EXTRA ANALYSES. IN FIRST ANALYSIS DELETE ROWS 2 AND 7 BY HAND. IN SECOND DELETE 1 AND 2, IN THIRD DELETE 7 AND 8. THE ROW SCORES OF THESE THREE ANALYSES ARE SAVED BY HAND IN THE .SAV-FILE STABILITY.SAV. CORRELATIONS BETWEEN THESE LAST THREE COLUMNS AND THE FIRST COLUMNS ARE REPORTED IN PAPER.
