# Supplementary material for: Doing Philosophy Effectively: Student Learning in Classroom Teaching
Source: PLoS One. 2015 Sep 17;10(9):e0137590. doi: 10.1371/journal.pone.0137590 (PMC4574705; doi:10.1371/journal.pone.0137590)
Supplement: S4 File — (DOCX) [file pone.0137590.s004.docx]

**Supporting Information**

**S6 File**

**Correspondence analysis (CA), interpretation**

CA of the super-indicator matrix can be described in multiple ways. One way is as a PCA for categorical variables. This approach is used by SPSS where CA of the super-indicator matrix is presented under the name CATPCA (see also [1]). Comparable to PCA, CA is a descriptive method for the data, not an inferential method. Also comparable to PCA is that in CA the aim is to summarize as much information in the data in a few dimensions, preferably one or two.

Another way to explain CA is as reciprocal averaging [1]. The idea is that in the final solution the rows (lessons) are in the weighted average of the columns (levels of the variables), and at the same time the columns are in the weighted average of the rows. Closely related is a geometrical explanation that is helpful in the interpretation. Rows are close together when they are similar in the sense that they use similar levels of the variables, and are further apart when they use different levels of the variables. So Lessons 1 and 2 are close together because they have many levels of the variables in common, and Lessons 2 and 7 are far apart because they have no levels of variables in common (see the super-indicator matrix in the manuscript). Levels of variables are close together when they are used by the same rows (lessons) and they are further apart when they are used by different rows. For example, Highest level 5 is close to MA philosophy is yes because they are used by similar lessons, and Highest level 4 and MA philosophy is yes are far apart because they do not have many lessons in common. Many details on the geometric interpretation are well described by Greenacre [2]. There are also relations to modelling, such as to loglinear modelling [3] and latent class modelling [4].

For further details to the construction of the super-indicator matrix we refer to S7 File. This document also relates CA to a set of two-way contingency tables of all pairs of variables that constitute the super-indicator matrix.

**References**

[1] Gifi A (1990) Nonlinear Multivariate Analysis*.* New Jersey: Wiley.

[2] Greenacre M (2007) Correspondence Analysis in Practice. Boca Raton, CRC, second edition.

[3] Van der Heijden PGM, de Falguerolles A, de Leeuw J (1989). A Combined Approach to Contingency Table Analysis Using Correspondence Analysis and Loglinear Analysis. Applied Statistics 38, 249-92.

[4] Van der Heijden PGM, Gilula Z, van der Ark LA (1999) An Extended Study into the Relationships between Correspondence Analysis and Latent Class Analysis. In: Sobel M and Becker M (eds) Sociological Methodology 1999. Cambridge: Blackwell, 147-86.
