# Supplementary material for: Doing Philosophy Effectively: Student Learning in Classroom Teaching
Source: PLoS One. 2015 Sep 17;10(9):e0137590. doi: 10.1371/journal.pone.0137590 (PMC4574705; doi:10.1371/journal.pone.0137590)
Supplement: S6 File — (DOCX) [file pone.0137590.s006.docx]

**Supporting Information**

**S8 File**

**Correspondence analysis (CA), contributions of variables**

For each the categories separately it is possible to calculate the contribution to the first dimension. This contribution is a function of the squared coordinate and the number of lessons that has used the category. By adding the contributions of the categories that belong to the same variable, the contribution of this variable is obtained. Such contributions are part of the default output of most computer programs for CA. The contributions for each of the variables are

|  | Contributions |
| --- | --- |
| Approaches | .144 |
| Domains | .086 |
| Aim | .068 |
| Ma Phil. | .064 |
| Exp. After training | .133 |
| St. grade | .013 |
| # Teaching styles | .027 |
| Dialogue | .120 |
| Guidance | .095 |
| # Pearls | .010 |
| Duration (%) | .091 |
| Highest level | .120 |
| Methods of ccf | .025 |

There are 13 variables where the variable Approaches was used thrice (compare super-indicator matrix). Thus the average contribution is 1/15 = .067. This shows that the variables Domains, Aim, Experience after training, Dialogue, Guidance, Duration and Highest level contribute more than average.
