# Supplementary material for: Doing Philosophy Effectively: Student Learning in Classroom Teaching
Source: PLoS One. 2015 Sep 17;10(9):e0137590. doi: 10.1371/journal.pone.0137590 (PMC4574705; doi:10.1371/journal.pone.0137590)
Supplement: S7 File — (DOCX) [file pone.0137590.s007.docx]

**Supporting Information**

**S9 File**

**Correspondence analysis (CA), stability**

When we number the variables in the super-indicator matrix from 1 to 13, we report the correlations between the rows scores of the full analysis with the analysis minus variable 1 – 13 as

| Minus  variable | Correlation |
| --- | --- |
| 1 | .997 |
| 2 | .994 |
| 3 | .994 |
| 4 | .995 |
| 5 | .997 |
| 6 | .992 |
| 7 | .999 |
| 8 | .998 |
| 9 | .998 |
| 10 | .999 |
| 11 | .992 |
| 12 | .994 |
| 13 | .999 |

The analysis where three variables are left out (see manuscript) is .955. The row scores on which these correlations are based are in S5 Table.

We also studied the stability of the CA solution when the number of lessons was reduced. For this we eliminated pairs of rows. We chose the pairs of rows that were furthest away from the mean (of 0) as these will have the largest possible impact on the first dimension. These pairs are 2 and 7 (one most extreme on left and one most extreme on right of first dimension), 1 and 2 (two lessons most extreme on the left) and 7 and 8 (two lessons most extreme on the right. Correlations are calculated between the six row scores found by analyzing the reduced matrix and the corresponding six row scores in the original matrix (compare lowest line in Fig 4 in manuscript). The correlations are again very high, providing additional proof that the solution is very stable.

| Minus  cases | Correlation |
| --- | --- |
| 2 and 7 | .990 |
| 1 and 2 | .988 |
| 7 and 8 | .981 |

The row scores on which these correlations are based are in S5 Table.
